# Use of Indocyanine Green to Detect Intraoperative Staple-Line Leaks in Robotic Bariatric Surgery: An Observational Cohort Study

**DOI:** 10.7759/cureus.56991

**Published:** 2024-03-26

**Authors:** Muzi Meng, Jaskaran Saini, David Fan, Ajit Singh, Daniel T Farkas

**Affiliations:** 1 School of Medicine, American University of the Carribean, Cupecoy, SXM; 2 General Surgery, BronxCare Health System, Bronx, USA; 3 Surgery, BronxCare Health System, Bronx, USA

**Keywords:** intraoperative leak, bariatric surgery, robotic gastric bypass, robotic sleeve gastrectomy, staple-line leak, indocyanine green (icg)

## Abstract

Background

Bariatric surgeries aid weight loss in patients with morbid obesity, yet staple-line leaks pose safety concerns. Multiple methods are used to help identify these links. Intraluminal indocyanine green (ICG) has been shown to be useful in other applications, and its use in robotic bariatric surgeries is underexplored.

Objective

The primary objective of this research project was to demonstrate the usage of intraluminal ICG in detecting staple-line leaks during robotic sleeve gastrectomy and robotic gastric bypass.

Settings

The research was conducted at Bronxcare Health System between June 2022 and June 2023.

Methods

We studied 150 consecutive participants undergoing robotic sleeve gastrectomy or robotic gastric bypass. Intraluminal ICG was used in each case in order to identify leaks. Data on comorbidities, detected intraoperative leaks, and detected postoperative leaks were collected.

Results

Out of the 150 patients who underwent robotic bariatric surgeries (139 for sleeve gastrectomy and 11 for gastric bypass), four cases (two for each procedure) were identified with intraoperative leaks using ICG, resulting in an overall 2.66% incidence rate. In those four patients with intraoperative leaks, reinforcing sutures and a drain were placed. Following the surgeries, none of the patients had radiologic or clinical leaks identified.

Conclusions

Intraluminal ICG presents a novel approach for detecting staple-line leaks in robotic bariatric surgery. Future studies can be done to look at a larger series of patients and compare leak detection rates between ICG and other methods.

## Introduction

Bariatric surgery is a common method for achieving long-term weight loss in patients with obesity, and the robotic approach has gained popularity due to its minimally invasive nature and reduced postoperative complications [1,2]. Leak from a staple line is a major concern following bariatric surgery, as it can result in significant morbidity and mortality [3-5]. Postoperative leak often has a delayed manifestation [6] and can quickly lead to sepsis, peritonitis, and even death [7,8]. Timely detection and management of this complication are crucial to prevent complications [3-5]. Thereby, methods for preventing and identifying leaks are a prominent focus of current investigation [9]. Indocyanine green (ICG), a dye used in surgical procedures, offers a means to visualize tissue perfusion and identify leaks [10].

While various research studies have investigated the use of intraluminal ICG fluorescence to detect staple-line leaks in bariatric surgery patients, both during the surgery and in the postoperative period, the efficacy of this modality has not been specifically established for robotic bariatric surgery [10-12]. Robotic bariatric surgeries are able to provide surgeons with better vision as they can get up close and observe in 3D [1,2]. The use of ICG in robotic surgeries, as compared to laparoscopic surgeries, can assist in the identification of leaks that might have been missed due to better vision. This study, therefore, aims to evaluate the intraoperative usage of ICG in patients undergoing robotic sleeve gastrectomy or robotic gastric bypass [1,2].

## Materials and methods

Study design

This is a retrospective observational study designed to evaluate the use of intraluminal ICG for the detection of intraoperative leaks in the setting of robotic-assisted bariatric surgery. The study was performed at BronxCare Health between June 2022 and June 2023. This study recruited 150 consecutive patients meeting study eligibility criteria. Patient data was collected both intraoperatively and for six weeks of postoperative follow-up. The primary outcome assessed was the incidence of either intraoperative or postoperative leak. 

Inclusion and exclusion criteria

All patients who underwent bariatric surgery were initially included. A majority of patients had multiple comorbidities, data for which was collected before the surgical procedure. Subjects with a known allergy to ICG, severe renal impairment, or pregnant or breastfeeding women were excluded from the research process. Patients who were undertaking concomitant medications, below 18 or above 65 years of age, or who were active smokers were also excluded. Institutional Review Board (IRB) approval was obtained.

Leak detection/ICG technique 

**Figure 1:**
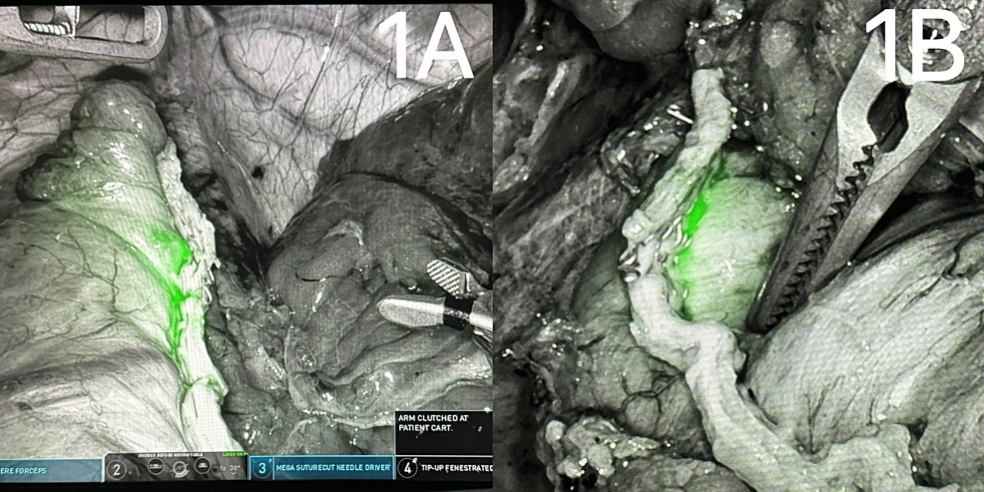
ICG Positive for Leak Positive leak study showing extravasation of fluorescent green fluid seen using Firefly laser technology. ICG: Indocyanine green

The overall surgical technique was left to the discretion and expertise of the performing surgeon. Following completion of the stapling or anastomosis, the leak test was done. 7 ml of ICG was mixed with 100 ml sterile water. This was instilled through the 40 French VisiGi bougie by the anesthesia team. Firefly technology on the DaVinci Xi robot was then utilized and the entire staple line or anastomosis was thoroughly evaluated. Firefly technology uses a near-infrared fluorescent camera, under which the ICG appears with a conspicuously bright green signal. A positive leak test was defined as visualized extravasation of fluorescent green fluid (Figure 1). Any identified leaks were reinforced with additional sutures in that area and a 19 French Blake Drain was left in place. All patients who had intraoperative leaks underwent an upper GI contrast study on postoperative day 1. Following a negative contrast study, they were started on fluids dyed with food coloring, to evaluate for any evidence of food coloring identified in their drains. Patients without an intraoperative leak identified underwent a contrast study per the choice of the operating surgeon, but it was not routine. All patients continued with the normal postoperative protocol and were discharged home when tolerating liquids without any complications.

Data analysis

Data on a number of variables was collected from patients including demographic variables of gender and age, height, initial weight and body mass index (BMI), comorbidities, past surgical history, and the use of tobacco, alcohol, or drugs. Descriptive statistics, including mean, standard deviation, and ranges for several continuous variables including age, and BMI. All patient information was allocated and stored on a secure server utilizing a Microsoft Excel Spreadsheet (Microsoft Corporation, Seattle, WA). For categorical variables, frequencies and proportions were calculated to determine the ratio of males and females in the study. The primary outcome of interest was the identification of intraoperative leaks identified by ICG, and postoperative leaks identified either radiologically or clinically. 

## Results

There were 150 consecutive patients undergoing robotic bariatric surgery, either robotic sleeve gastrectomy or robotic gastric bypass. Background variables such as age, BMI, medical comorbidities, and the individuals’ alcohol, smoking, and drug use were similar between the patients undergoing robotic sleeve gastrectomy and robotic gastric bypass (Table 1). With respect to gender, 125 (83.33%) were female, with the remaining 25 (16.67%) being male. Patients undergoing robotic bariatric surgery had an average age of 40.04 years and an average BMI of 42.85 kg/m^2^. 

**Table 1:** Population Characteristics for the Study Patient Population (n=150).

Background Variables	Robotic Sleeve (n = 139)	Robotic Gastric Bypass (n = 11)	Total (n = 150)
Age	40.08 + 10.76 (18-63)	39.55 + 9.71 (26-57)	40.04 + 10.66 (18-63)
BMI	42.71 + 5.48 (35-60)	44.62 + 5.27 (38-53)	42.85 + 5.47 (35-60)
Gender	Males: 25 (17.98%) Females: 114 (82.01%)	Males: 0 (0.00%) Females: 11 (100.00%)	Males: 25 (16.67%) Females: 125 (83.33%)
A summary of study sample (N=150) characteristics. Summary statistics are provided in either count (proportion) or mean + SD (Range) format. The appropriate one is used for each characteristic listed within the table. Parentheses proportions are representative of only responses and exclude missing responses. Age represented in years; SD standard deviation represented in years; BMI body mass index represented in kg/m^2^.			

Patient population and key descriptives

Out of 150 total patients, 4 (2.66%) had an intraoperative leak identified. All the leaks were extremely tiny streams that were not visible with regular light and could only be seen using the firefly technology. In each case, the area where the leak was seen was reinforced with more sutures, and a drain was placed. All patients with leaks had an upper GI contrast study with gastrografin on postoperative day 1, and all were negative. All these patients were started on liquids that contained food coloring, and none had food coloring appear in the drain. They all were followed in the clinic following the usual postoperative protocol and had similar outcomes to the overall study population. No patients in the study population had a postoperative leak identified either radiologically or clinically. There were no complications related to the use of ICG. 

## Discussion

Several studies have evaluated the effectiveness of using intraluminal ICG in laparoscopic bariatric surgery. Pavone et al. investigated its intraoperative use in identifying leak sites, finding that it accurately identifies leak sites, allowing for timely intervention and reducing overall postoperative morbidity [10]. Kalmar et al. conducted a comparative study on laparoscopic sleeve gastrectomy, evaluating the effectiveness of ICG compared to methylene blue dye in detecting postoperative leaks [11]. The study concluded that ICG surpasses methylene blue dye in terms of accuracy and precision, recommending its use in laparoscopic sleeve gastrectomy [11]. Another comparative study was conducted by Balla et al. which compared its usage with other traditional methods of leak detection [12]. Similar to other studies, they found that ICG is more accurate than conventional methods while having the potential to reduce morbidity [12]. Ortega et al. also examined the effectiveness of ICG in the identification of leaks during various bariatric surgeries and recommended its usage over other traditional methods [13]. A systematic review of five studies evaluated the use of ICG for the detection of leaks during bariatric surgery [14]. The authors concluded that ICG is a safe and effective method for the detection of intraoperative leaks, reducing the rate of re-operation and potentially the length of stay [14].

This study demonstrates the feasibility of using intraluminal ICG to detect leaks during robotic bariatric surgery. While this has been seen before in laparoscopic surgery [11,14], its use in robotic surgery has not been shown before. Using it during robotic surgery can be even more helpful, as the robot offers a lot of advantages. Firstly, the firefly technology is built into the system. Second, the robot camera offers improved vision due to its stereoscopic 3D vision, and the ability to get a magnified view. This allows the operator to identify even very tiny leaks, as were seen in our study.

In addition, another advantage of using ICG is that the dye is not visualized once the laser is disengaged, allowing the surgeon to perform the repair with an unaltered view of the patient's anatomy [10]. The use of intraluminal ICG for the detection of intraoperative and postoperative leaks is a relatively new method due to which studies should also focus on any adverse events that might be unique to this method [15].

Given the significant complications caused by a staple-line leak in bariatric surgery, researchers have tested various modalities that can be useful in the identification of leak sites, either intraoperatively or postoperatively. One such method is the use of intraoperative endoscopy which primarily involves an endoscopist assisting the surgeons with the identification of leak sites [16]. Though this method is effective in the identification of intraoperative leaks [16], Kalmar et al. noted that using an additional endoscopist increases the time and cost of the surgical procedure [10]. Using the ICG technique, the water is instilled by the anesthesia team, and no extra proceduralist is necessary. Another method is the use of methylene blue to detect intraoperative and post-operative leaks. Some studies have highlighted that this method has a higher false negative rate [17,18]. A downside to this method is that if there is a leak, the dark color of the methylene blue stains the surrounding tissue, and results in reduced visibility for the surgeon to continue operating in this region. Which is now critical as the leak needs to be repaired. The use of ICG is advantageous here, as it is invisible when not using the near-infrared fluorescent camera.

Another technique is the air leak test, where air is insufflated into the stomach while it is under saline. A leak is identified by the leakage of bubbles. However, a problem with this technique is that small leaks can be missed [12]. The surgeon needs to see the bubbles come out just as they happen. With ICG there is more time for the ICG to come out over time, which can help identify a small leak. 

**Figure 2:**
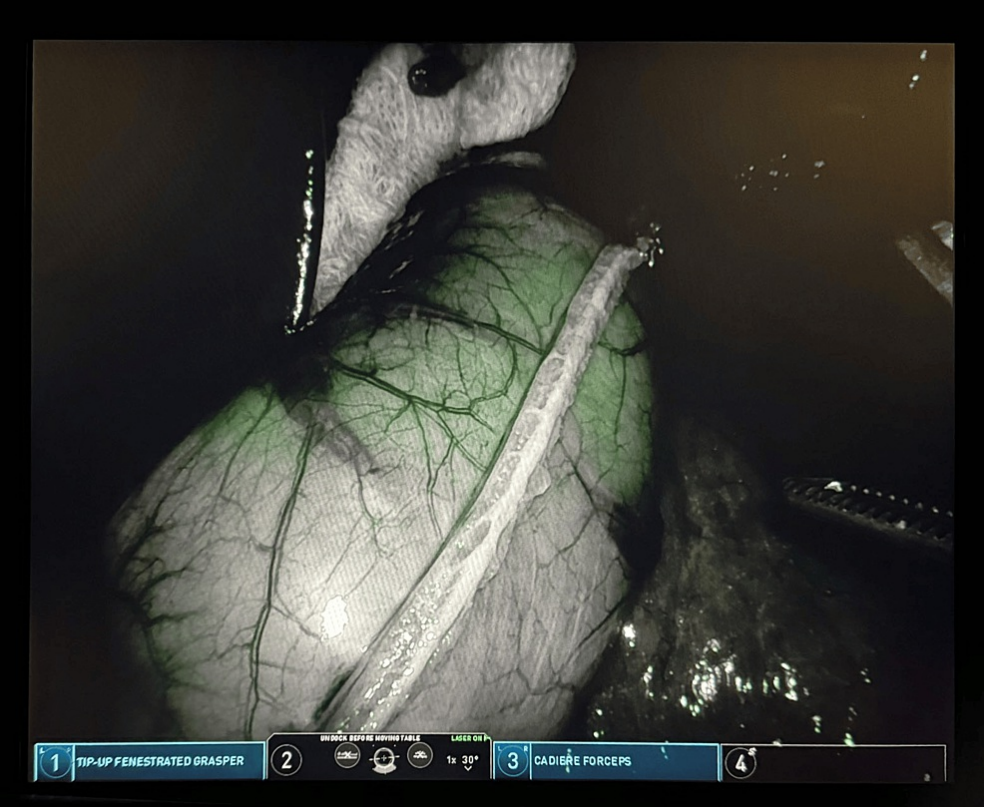
ICG Negative for Leak Negative leak study showing only dull green glowing through the wall, but without extravasation of fluorescent green fluid. ICG: Indocyanine green

Other studies have highlighted that one of the major limitations of using intraluminal ICG fluorescence for the detection of leak sites is the high incidence of false positives; reported as high as 10-20% in some studies [19,20]. Our study did not find that to be the case. It is critical to clearly define what constitutes a positive leak. Frequently, the fluorescence can be seen as glowing ‘dull’ through the stomach wall, and this should not be interpreted as a staple-line leak (Figure 2) [10]. Surgeons should assess for the actual flow of neon extravasation and if this is not visible, the ICG leak test should be considered negative. With increased operator experience, distinguishing between true positives and dull glow false positives becomes more distinct, leading to a very low false positive rate.

One limitation of our study is the low overall number of leaks. While this is typical in bariatric surgery, our study did however identify four intraoperative leaks, which demonstrates that this method of detection works. There were no postoperative leaks in either group of patients, neither the ones with intraoperative leaks nor the ones without. However, this can be attributed to the fact that all the intraoperatively identified leaks were addressed and repaired at the time of identification. Another limitation is that we did not use an alternative method of detecting leaks, to compare with ICG findings. While this would be a useful future project, we can say that no patients had a postoperative leak identified, so there was no false negative ICG result. 

An additional limitation of intraluminal ICG, which is common to the other intraoperative modalities, is its inability to assess vascular supply. In fact, ICG can be given intravascularly in order to assess perfusion. This has been used in other types of surgeries [19]. However, this is not the typical mechanism of leak in bariatric surgery and was not evaluated in our study.

Finally, all the leaks identified in our study were very tiny. We feel it’s likely they wouldn’t have been seen using alternative methods. It’s possible that none of these leaks would have ended up having clinical significance. However, given that leaks can have such consequential impacts on the outcomes, we are comfortable using an overly sensitive leak-detecting modality. Especially if it does not require much additional cost, time, or personnel.

## Conclusions

ICG emerges as a pivotal technique for identifying staple-line leaks during robotic bariatric surgery, offering significant advancements in surgical precision and patient safety by providing unparalleled visualization capabilities and eliminating the need for additional personnel like a separate endoscopist. Its integration heralds a paradigm shift in intraoperative leak detection, promising enhanced accuracy and efficiency, enabling proactive identification and immediate intervention in case of leaks, thereby minimizing postoperative complications and optimizing resource utilization. Future comparative studies are imperative to validate ICG's clinical efficacy and diagnostic performance and provide a more investigative quantitative analysis. This will help standardize across diverse patient populations and elevate the standard of care in robotic bariatric surgery. Moreover, the adoption of ICG holds profound implications for surgical innovation and healthcare delivery, showcasing the transformative potential of technology in enhancing surgical outcomes and patient care through interdisciplinary collaboration and continued technological innovation.

## References

[1] Bhatia P, Bindal V, Singh R, Gonzalez-Heredia R, Kalhan S, Khetan M, John S (2014). Robot-assisted sleeve gastrectomy in morbidly obese versus super obese patients. JSLS.

[2] Bindal V, Bhatia P, Dudeja U, Kalhan S, Khetan M, John S, Wadhera S (2015). Review of contemporary role of robotics in bariatric surgery. J Minim Access Surg.

[3] Silecchia G, Iossa A (2018). Complications of staple line and anastomoses following laparoscopic bariatric surgery. Ann Gastroenterol.

[4] Praveenraj P, Gomes RM, Kumar S, Senthilnathan P, Parthasarathi R, Rajapandian S, Palanivelu C (2016). Management of gastric leaks after laparoscopic sleeve gastrectomy for morbid obesity: a tertiary care experience and design of a management algorithm. J Minim Access Surg.

[5] Loo GH, Rajan R, Nik Mahmood NR (2019). Staple-line leak post primary sleeve gastrectomy. A two patient case series and literature review. Ann Med Surg (Lond).

[6] Mohamed AA, Humaida AA, Qureshi AS (2021). Delayed post-laparoscopic sleeve gastrectomy leak successfully treated with endoscopic clips and tissue adhesive: case report and literature review. Cureus.

[7] Benedix F, Benedix DD, Knoll C, Weiner R, Bruns C, Manger T, Stroh C (2014). Are there risk factors that increase the rate of staple line leakage in patients undergoing primary sleeve gastrectomy for morbid obesity?. Obes Surg.

[8] Boeker C, Schneider B, Markov V (2021). Primary sleeve gastrectomy and leaks: the impact of fundus-wall thickness and staple heights on leakage-an observational study of 500 patients. Front Surg.

[9] Bekheit M, Katri KM, Nabil W, Sharaan MA, El Kayal ES (2013). Earliest signs and management of leak after bariatric surgeries: single institute experience. Alex J Med.

[10] Pavone G, Fersini A, Pacilli M, Cianci P, Ambrosi A, Tartaglia N (2022). Anastomotic leak test using indocyanine green during laparoscopic Roux-en-Y gastric bypass: a cohort study. Ann Med Surg (Lond).

[11] Kalmar CL, Reed CM, Peery CL, Salzberg AD (2020). Intraluminal indocyanine green for intraoperative staple line leak testing in bariatric surgery. Surg Endosc.

[12] Balla A, Corallino D, Quaresima S, Palmieri L, Meoli F, Cordova Herencia I, Paganini AM (2022). Indocyanine green fluorescence angiography during laparoscopic bariatric surgery: a pilot study. Front Surg.

[13] Ortega CB, Guerron AD, Yoo JS (2018). The use of fluorescence angiography during laparoscopic sleeve gastrectomy. JSLS.

[14] Carrano FM, Di Lorenzo N (2020). The use of indocyanine green in bariatric surgery: a systematic review. J Gastric Surg.

[15] Hope-Ross M, Yannuzzi LA, Gragoudas ES (1994). Adverse reactions due to indocyanine green. Ophthalmol.

[16] Chen IS, Tsai MS, Chen JH, Chen CY, Chen IL, Tai CM (2022). The utility of intraoperative endoscopy to assist novice surgeons in the detection of gastric stenosis during laparoscopic sleeve gastrectomy. BMC Surg.

[17] Kirby GC, Macano CA, Nyasavajjala SM (2017). The Birmingham experience of high-pressure methylene blue dye test during primary and revisional bariatric surgery: a retrospective cohort study. Ann Med Surg (Lond).

[18] Bingham J, Lallemand M, Barron M, Kuckelman J, Carter P, Blair K, Martin M (2016). Routine intraoperative leak testing for sleeve gastrectomy: is the leak test full of hot air?. Am J Surg.

[19] Cho YJ, Namgoong JM, Kwon HH, Kwon YJ, Kim DY, Kim SC (2021). The advantages of indocyanine green fluorescence imaging in detecting and treating pediatric hepatoblastoma: a preliminary experience. Front Pediatr.

[20] Kitagawa N, Shinkai M, Mochizuki K (2015). Navigation using indocyanine green fluorescence imaging for hepatoblastoma pulmonary metastases surgery. Pediatr Surg Int.

